# Functional brain networks in the schizophrenia spectrum and bipolar disorder with psychosis

**DOI:** 10.1038/s41537-020-00111-6

**Published:** 2020-09-02

**Authors:** Edwin van Dellen, Corinna Börner, Maya Schutte, Simone van Montfort, Lucija Abramovic, Marco P. Boks, Wiepke Cahn, Neeltje van Haren, René Mandl, Cornelis J. Stam, Iris Sommer

**Affiliations:** 1 Department of Psychiatry, Brain Center, University Medical Center Utrecht, Utrecht, The Netherlands; 2 Department of Intensive Care Medicine and UMC Utrecht Brain Center, University Medical Center Utrecht, Utrecht University, Utrecht, The Netherlands; 3 University of Groningen, Department of Neuroscience, University Medical Center Groningen, Groningen, The Netherlands; 4 Department of Child and Adolescent Psychiatry/Psychology, Erasmus Medical Center, Sophia Children’s Hospital, Rotterdam, The Netherlands; 5 Department of Clinical Neurophysiology and MEG Center, Amsterdam University Medical Center, Amsterdam, The Netherlands; 6 Department of Biological and Medical Psychology, University of Bergen, Bergen, Norway

**Keywords:** Psychosis, Psychosis

## Abstract

Psychotic experiences have been proposed to lie on a spectrum, ranging from subclinical experiences to treatment-resistant schizophrenia. We aimed to characterize functional connectivity and brain network characteristics in relation to the schizophrenia spectrum and bipolar disorder with psychosis to disentangle neural correlates to psychosis. Additionally, we studied antipsychotic medication and lithium effects on network characteristics. We analyzed functional connectivity strength and network topology in 487 resting-state functional MRI scans of individuals with schizophrenia spectrum disorder (SCZ), bipolar disorder with a history of psychotic experiences (BD), treatment-naïve subclinical psychosis (SCP), and healthy controls (HC). Since differences in connectivity strength may confound group comparisons of brain network topology, we analyzed characteristics of the minimum spanning tree (MST), a relatively unbiased backbone of the network. SCZ and SCP subjects had a lower connectivity strength than BD and HC individuals but showed no differences in network topology. In contrast, BD patients showed a less integrated network topology but no disturbances in connectivity strength. No differences in outcome measures were found between SCP and SCZ, or between BD patients that used antipsychotic medication or lithium and those that did not. We conclude that functional networks in patients prone to psychosis have different signatures for chronic SCZ patients and SCP compared to euthymic BD patients, with a limited role for medication. Connectivity strength effects may have confounded previous studies, as no functional network alterations were found in SCZ after strict correction for connectivity strength.

## Introduction

Psychosis is defined by the experience of hallucinations, delusions, and the disorganization of thought and speech^1^. Psychotic experiences are a core feature of schizophrenia spectrum disorders (SCZ) but are also highly prevalent in bipolar disorder (BD)^2–4^, and in attenuated form in the healthy population (subclinical psychosis, SCP). As such, psychosis has been proposed to lie on a spectrum, which ranks these experiences regarding their severity in clinical and subclinical populations^5^. Even though psychotic experiences in SCZ and BD share phenomenological features^4,6,7^, it remains unclear to what extent psychosis in BD shares a common pathophysiology with SCP and SCZ. Disentangling the pathophysiology underlying psychosis in SCP, SCZ, and BD may help to tailor psychosis treatment based on biological features.

Neuroimaging studies have provided evidence for the disconnection hypothesis in SCZ stating that psychosis in SCZ can be understood from aberrant anatomical connections and functional interactions in the brain^8,9^. Network theory provides a framework to study structural or functional connections between brain areas on a systems level, where network nodes represent brain regions and edges reflect axonal (as measured with diffusion tensor imaging) or functional connections between nodes^10^. Several characteristics have been defined to quantitatively describe the organization of networks, including the strength or number of connections that are present (i.e., connectivity strength), the number of steps it takes to travel across the network (i.e., path length, a measure of efficiency), and the presence of highly connected, central hub regions; the so-called rich club^11^.

In structural brain networks of clinical and subclinical subjects with psychosis, connectivity strength is generally decreased as compared to controls^12–16^. Analysis of network topology characteristics such as efficiency, however, has shown mixed or even contradicting results^15,17–19^. In functional networks of subjects with psychosis, both increases and decreases of connectivity strength have been reported, as well as mixed results on network characteristics^12,14,20,21^. Similarly, studies in BD have not yet led to consensus on connectivity alterations related to the disorder^22–26^. Clinical explanations for the variability of findings in brain network studies may include differences in studied populations, disease stages, and medication effects. However, increasing evidence shows that methodological choices during the imaging data analysis, ranging from data acquisition to the definition of network characteristics, may have considerable impact on group comparisons^21,27^.

**Fig. 1:**
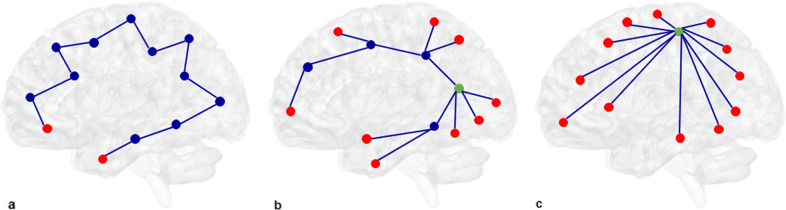
MST visualizations. **a**–**c** Brain regions and their connections are visualized as nodes (circles) and edges (lines). Red nodes are leaf nodes with only one connection. **a** Path-like MST with a diameter of twelve edges. **b** Intermediate between a path-like and a star-like MST with a diameter of six edges. The green node has a higher degree (five connecting edges) than the red leaf nodes. **c** Star-like MST: The green node is a node with high betweenness centrality: When connecting any two nodes in the network you have to pass the green node. Kappa, a measure that characterizes the degree distribution, is highest for **a**, intermediate for **b**, and lowest for **c**.

We studied fMRI connectivity and network topology in a large sample of individuals with SCZ, SCP, BD, and healthy controls (HC), using strict correction for confounders in network analysis, and exploring possible effects of antipsychotic medication. We analyzed resting-state fMRI scans and applied the minimum spanning tree (MST) analysis to study functional network topology. The MST is a backbone of the network that is relatively insensitive to differences in connectivity strength, which often confound group comparisons of brain network topology, while MST characteristics can be interpreted along the lines of conventional network characteristics^27–29^ (Fig. 1; Table 1). We analyzed whether connectivity strength differences can be disentangled from network topology differences in fMRI functional networks of individuals with a history of psychotic experiences. In particular, we compared SCP, SCZ, BD with a history of psychotic episodes, and HC. To investigate possible medication effects on functional connectivity and network topology we compared (1) medication-naïve SCP individuals and SCZ patients (mostly) using antipsychotic medication, and (2) BD patients that used antipsychotics and those that did not.

**Table 1:** Explanations of minimum spanning tree measures (Stam et al.^27^; Tewarie et al.^29^).

MST Measures	Definition	Interpretation
Connectivity strength (mean edge)	Mean of edge weights in MST network	Connectivity strength
Overlap	Fraction of edges that two MSTs have in common	Overlap ranges from 0 (no overlapping edges) to 1 (exact match of MSTs)
Diameter	Longest distance between two most remote nodes (expressed in number of edges)	Indication of network efficiency. A low diameter means that information is efficiently processed between remote brain regions.
Degree divergence/Kappa	Measure of the broadness of the degree distribution	Measure of degree diversity across nodes and importance of hubs. A high kappa indicates a broader degree distribution and thereby more hubs are expected. Kappa is related to resilience against attacks.
Leaf fraction	Fraction of leaf nodes (nodes with only one connection) in the MST	Measure of network centrality, integration, and efficiency. A high leaf fraction means that the network is largely dependent on central nodes.
Degree	Number of edges connected to a given node	Measure of regional importance. Nodes with a high degree may be hub nodes and are more important in the network.
Betweenness centrality of node X	Fraction of shortest paths between any two nodes y and z that are passing through x but not including x	Measure of network centrality (how central a node’s role is in the overall network communication). When connecting any two nodes in the network how likely is it to pass node X? Betweenness centrality ranges from 0 (leaf node) to 1 (central node in a star-like network).

## Results

### Subject characteristics

Subject characteristics are summarized in Table 2. Groups significantly differed on age (*F*(3, 483) = 24.02, *p* < 0.001), sex (*X²*(3) = 20.09, *p* < 0.001), and education (*F*(3, 483) = 9.74, *p* < 0.001; see Supplementary Materials: Subject Characteristics). Motion parameters also differed between the groups (*F*(3, 486) = 21.586, *p* < 0.001) with SCZ (M = 0.10, SD = 0.039) and BD patients (M = 0.10, SD = 0.032) showing more movement during scanning than SCP individuals (M = 0.08, SD = 0.029) and HC (M = 0.08, SD = 0.026). However, this was of little influence on connectivity measures after strict motion correction (see Supplementary Fig. 1).

**Table 2:** Characteristics of the participant population.

	HC	SCP	SCZ	BD
Sample size	219	35	97	136
Sex % (*n*)*				
Female	46.1% (101)	74.3% (26)	32% (31)	50.7% (69)
Male	53.9% (118)	25.7% (9)	68% (66)	49.3% (67)
Age (years)*				
Mean Age (SD)	40.76 (14.532)	42.09 (15.038)	31.45 (10.551)	46.12 (11.651)
Age range (min–max)	19–78	18–65	18–74	21–73
Handedness % (*n*)				
Right	83% (182)	74% (26)	83% (80)	87% (118)
Left	16% (36)	26% (9)	8% (8)	13 % (18)
Bilateral/N/A	1% (1)	0% (0)	9% (9)	0% (0)
Education (years)*				
Mean years of education (SE)	14.03 (.184)	13.63 (.312)	12.21 (.407)	13.50 (.249)
Diagnosis % (*n*)				
No diagnosis	100% (219)	100% (35)	0% (0)	0% (0)
Schizophrenia	0% (0)	0% (0)	53.6% (52)	0% (0)
Schizophreniform disorder	0% (0)	0% (0)	1% (1)	0% (0)
Psychosis NOS	0% (0)	0% (0)	27.9% (27)	0% (0)
Bipolar-I disorder	0% (0)	0% (0)	0% (0)	100 % (136)
Missing	0% (0)	0% (0)	17.5% (17)	0% (0)
Hallucinations % (*n*)				
Present	0% (0)	100% (35)	82.5% (80)	52.2% (71)
Absent	98.2% (215)	0% (0)	10.3% (10)	47.8% (65)
Missing	1.8% (4)	0% (0)	7.2% (7)	0% (0)
Delusions % (n)				
Present	.5% (1)	34.3% (12)	68% (66)	92.6% (126)
Absent	97.3% (213)	57.1% (20)	5.2% (5)	7.4% (10)
N.A.	0% (0)	0% (0)	14.4% (14)	0% (0)
Missing	2.3 (5)	8.6% (3)	12.4% (12)	0% (0)
SPQ score			n/a	n/a
Total *N*	44	32		
Mean (SD)	7.3 (6.1)	1.2 (12)		
PANSS scores				
Total *N*			42	
PANSS total mean (SD)	n/a	n/a	63 (14)	n/a
PANSS positive mean (SD)	n/a	n/a	15 (4)	n/a
PANSS negative mean (SD)	n/a	n/a	15 (6)	n/a
PANSS general mean (SD)	n/a	n/a	32 (7)	n/a
Medication % (*n*/total *n*)				
Antidepressants*	0% (0/219)	5.7% (2/35)	26.3% (21/80)	22.1% (30/136)
Mood stabilizers*	0% (0/219)	0% (0/35)	0% (0/97)	62.5% (85/136)
Antipsychotics*	0% (0/219)	0% (0/35)	90.6% (77/85)	48.5% (66/136)

Group differences in continuous variables were tested with one-way ANOVAs and differences in dichotomous variables were tested with Chi-Square tests. Significant differences at *p* = 0.05 are marked with an asterisk (*). Subjects can be classified within several medication categories.

*HC* healthy controls, *SCP* subclinical psychosis, *SCZ* schizophrenia spectrum disorder, *BD* bipolar disorder with psychosis, *SPQ* schizotypo-personality questionnaire, *PANSS* positive and negative symptom scale.

### Group comparisons

Results are summarized in Table 3 (see Supplementary materials: section matrix visualizations and group comparisons and Supplementary Table 5 for post-hoc test results). SCZ and SCP subjects had lower connectivity strength than HC and BD individuals (*F*(3, 480) = 5.62, *p* = 0.001, *ƞ*^*2*^ = 0.034). No differences in global MST network topology were found between SCZ, SCP, and HC. Mean connectivity strength in BD patients did not significantly differ from HC. However, BD patients showed significantly lower kappa (less diversity in node degree; *F*(3, 480) = 4.09, *p* = 0.007, *ƞ*^*2*^ = 0.025) and leaf fraction scores (decreased network integration or efficiency; *F*(3, 480) = 3.49, *p* = 0.016, *ƞ*^*2*^ = 0.021) than HC and SCP individuals. We found a nonsignificant trend for a lower kappa (*p* = 0.058) and leaf fraction (*p* = 0.092) in BD compared to SCZ. Sex had a significant effect as covariate on connectivity strength (*F*(1, 480) = 5.850, *p* = 0.016, *ƞ*^*2*^ = 0.012), with higher connectivity strength in males. Age had a significant effect as covariate on kappa (*F*(1, 480) = 6.588, *p* = 0.011, *ƞ*^*2*^ = 0.014) and leaf fraction (*F*(1, 480) = 15.948, *p* < 0.001, *ƞ*^*2*^ = 0.032), with higher kappa and leaf fraction scores with increasing age.

**Table 3:** Group differences in minimum spanning tree measures.

	df	F	p	${\eta}^2$	Mean	SE
*Group differences in MST measures*						
Connectivity strength						
Age	1480	1.31	0.255	0.003		
Sex	1480	5.85	0.016*	0.012		
Education	1480	3.78	0.063	0.008		
Group	3480	5.62	0.001*	0.034		
HC					0.639	0.004
SCP					0.618	0.009
SCZ					0.616	0.006
BD					0.640	0.005
Diameter						
Group	3480	1.05	0.371	0.006		
HC					0.108	0.001
SCP					0.108	0.003
SCZ					0.111	0.002
BD					0.111	0.002
Kappa						
Age	1480	6.59	0.011*	0.014		
Sex	1480	1.33	0.249	0.003		
Education	1480	0.041	0.867	<0.001		
Group	3480	4.09	0.007*	0.025		
HC					2.840	0.008
SCP					2.867	0.020
SCZ					2.835	0.013
BD					2.802	0.011
Leaf fraction						
Age	1480	15.95	<0.001*	0.032		
Sex	1480	0.822	0.365	0.002		
Education	1480	0.492	0.515	0.001		
Group	3480	3.49	0.016*	0.021		
HC					0.472	0.002
SCP					0.478	0.004
SCZ					0.471	0.003
BD					0.465	0.002

Group differences were tested with ANCOVAs with age, sex, and education as covariates and the Benjamini–Hochberg FDR correction for multiple testing. Significant differences at *p* = 0.05 are marked with an asterisk (*).

*HC* healthy controls, *SCP* subclinical psychosis, *SCZ* schizophrenia spectrum disorder, *BD* bipolar disorder with psychosis.

### Sensitivity analysis

The replication of our findings with age-matched groups (see Supplementary Materials: Sensitivity Analysis and Supplementary Tables 6 and 7 for details) showed that all results from our main analysis were replicated except for the difference in connectivity strength between SCP and HC, which became nonsignificant, and the difference in leaf fraction between SCZ and BD, which became significant with age-matched groups. SCZ patients had lower connectivity strength than HC (*F*(1, 189) = 6.956, *p* = 0.010, *ƞ*^*2*^ = 0.035) and BD subjects (*F*(1, 117) = 8.143, *p* = 0.006, *ƞ*^*2*^ = 0.065). SCP subjects had lower connectivity strength than BD patients (*F*(1, 100) = 7.123, *p* = 0.009, *ƞ*^*2*^ = 0.066) but connectivity strength was no longer significantly lower in SCP relative to HC (*F*(1, 100) = 3.213, *p* = 0.076, *ƞ*^*2*^ = 0.031). No differences in global MST network topology were found between SCZ, SCP, and HC. Connectivity strength in BD patients did not significantly differ from HC. However, BD patients had significantly lower kappa scores (less diversity in node degree) than HC (*F*(1, 267) = 7.805, *p* = 0.006, *ƞ*^*2*^ = 0.028) and SCP (*F*(1, 100) = 7.779, *p* = 0.006, *ƞ*^*2*^ = 0.072) and lower leaf fraction scores (decreased network integration or efficiency) than HC (*F*(1, 267) = 5.163, *p* = 0.024, *ƞ*^*2*^ = 0.019), SCP (*F*(1, 100) = 7.701, *p* = 0.007, *ƞ*^*2*^ = 0.071), and SCZ (*F*(1, 117) = 4.801, *p* = 0.030, *ƞ*^*2*^ = 0.039).

### Regional network analysis

**Fig. 2:**
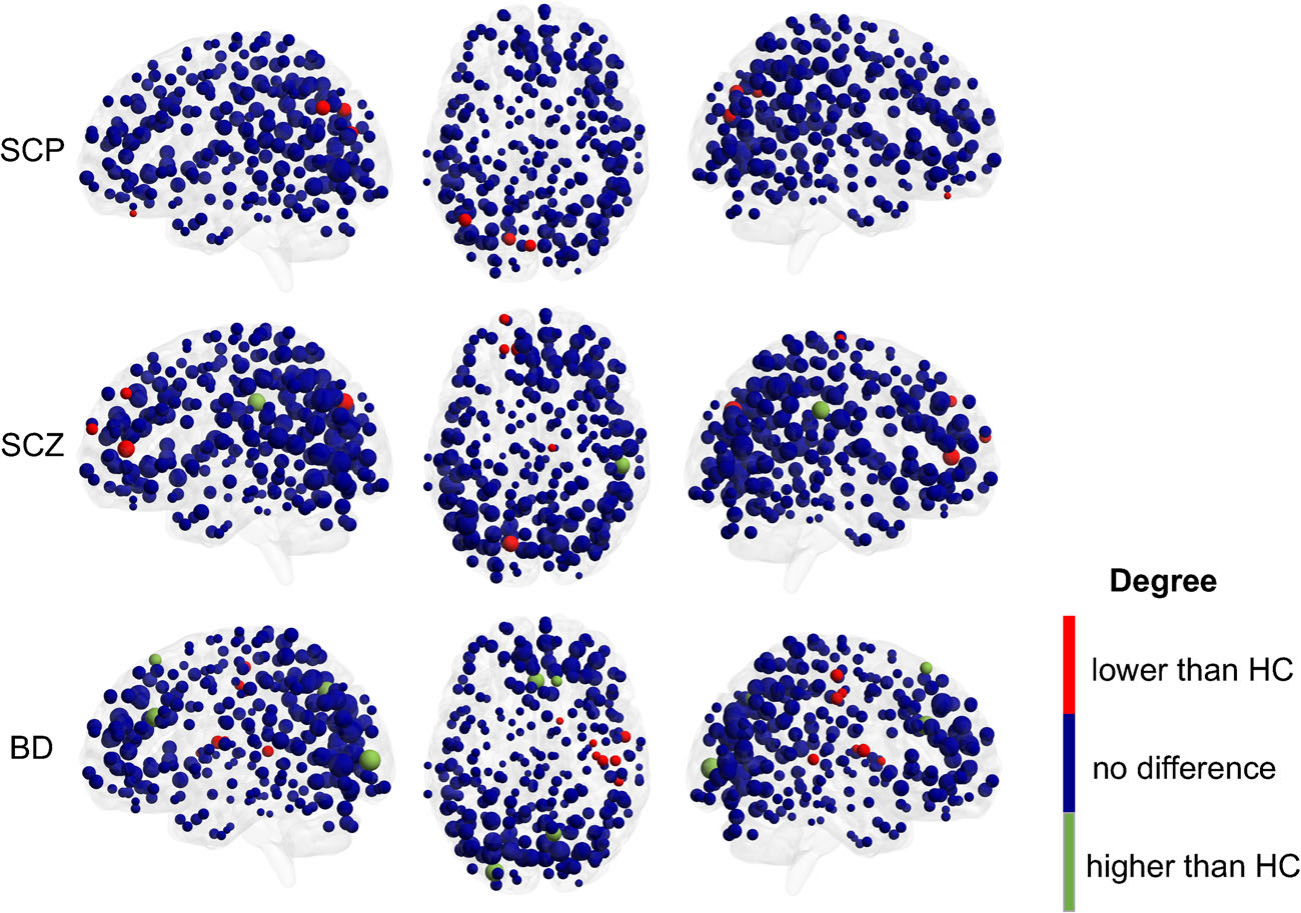
Visualization of regional degree differences between the networks of psychosis groups and age-matched healthy controls. The nodal size corresponds to their degree. Blue nodes mark regions that do not differ compared to healthy controls, while red nodes mark regions with a lower degree and green nodes mark regions with a higher degree compared to controls. HC healthy controls, SCP subclinical psychosis, SCZ schizophrenia spectrum disorder, BD bipolar disorder with psychosis.

Results are visualized in Fig. 2. Regional analyses (significant at *p* = 0.05 after correction for multiple comparisons) showed that left occipital regions have a lower degree in SCP and SCZ compared to HC. Additionally, left frontal regions have a lower degree while the right supramarginal gyrus has a higher degree in SCZ compared to HC. In contrast, bilateral frontal and occipital regions have a higher degree in BD relative to HC, while right temporal regions have a lower degree. Detailed results can be found in the supplementary material (Section regional network analysis, Supplementary Tables 8 and 9, Supplementary Figs. 2–4).

### Medication effects

Medication effects on MST measures were examined by comparing the differences on MST measures between (1) SCZ and SCP subjects and (2) BD patients with (*N* = 66) and without antipsychotic medication (*N* = 65) and (3) BD patients with (*N* = 85) and without (*N* = 51) lithium. Differences in global MST network topology were found neither between SCZ patients, medication-naïve SCP individuals, and HC, nor between BD patients that used antipsychotics and those that did not (Tables 3 and 4, Supplementary materials).

**Table 4:** Medication differences in minimum spanning tree measures.

	df	F	p		Mean	SE
*Antipsychotic medication effects on MST measures*						
Connectivity strength						
Group	1126	0.524	0.470			
BD/AP					0.641	0.006
BD/nAP					0.635	0.006
Diameter						
Group	1126	0.817	0.368			
BD/AP					0.112	0.002
BD/nAP					0.109	0.002
Kappa						
Group	1126	<0.001	0.990			
BD/AP					2.802	0.013
BD/nAP					2.802	0.013
Leaf fraction						
Group	1126	0.008	0.929			
BD/AP					0.467	0.003
BD/nAP					0.467	0.003
*Lithium effects on MST measures*						
Connectivity Strength						
Group	1131	3.702	0.057			
BD/li+					0.632	0.005
BD/li−					0.648	0.008
Diameter						
Group	1131	1.131	0.290			
BD/li+					0.109	0.002
BD/li−					0.113	0.002
Kappa						
Group	1131	0.972	0.326			
BD/li+					2.800	0.013
BD/li−					2.819	0.014
Leaf fraction						
Group	1131	0.150	0.699			
BD/li+					0.466	0.003
BD/li−					0.469	0.003

Group differences were tested with ANCOVAs with age, sex, and education as covariates and the Benjamini–Hochberg FDR correction for multiple testing.

*HC* healthy controls, *SCP* subclinical psychosis, *SCZ* schizophrenia spectrum disorder, *BD* bipolar disorder with psychosis, *BD/AP* bipolar disorder with antipsychotic use, *BD/nAP* bipolar disorder without antipsychotic use, *BD/li+* bipolar disorder with lithium use, *BD/li−* bipolar disorder without lithium use.

## Discussion

**Fig. 3:**
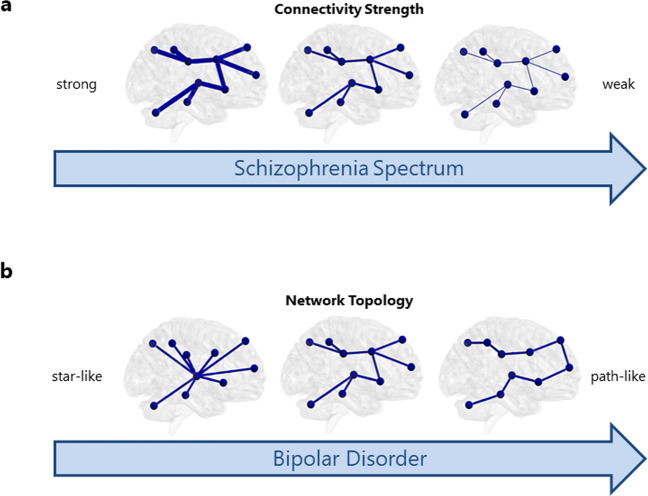
Different neural mechanisms to psychosis in the schizophrenia spectrum and bipolar disorder. **a** In the schizophrenia spectrum, the resting-state MRI network is characterized by decreased connectivity strength, while the topology of the network does not differ from controls. **b** In patients with bipolar disorder with a history of psychosis, the connectivity strength does not differ from controls, but the network topology is less integrated.

We aimed to characterize functional connectivity and brain network characteristics in relation to the schizophrenia spectrum and in bipolar disorder with psychosis to disentangle neural correlates to psychosis. We found that functional networks of SCZ and SCP individuals are characterized by decreased MST connectivity strength compared to HC (see Fig. 3a); however, no differences in network topology were found. Interestingly, SCP and SCZ individuals did not differ from each other. Functional networks of BD patients seem to be topologically different in that their networks are less centralized, less integrated, and less efficient compared to HC (see Fig. 3b) while no differences in connectivity strength were found. Our findings indicate that disconnection characterizes functional networks of both SCZ and BD patients with psychosis. However, in SCZ this might be due to a global decrease in connectivity strength, whereas network topological differences are found in BD compared to other groups. Importantly, this indicates that distinct neural correlates are related to psychosis in BD as compared to SCP and SCZ. Our study mostly included SCZ patients with chronic symptoms as well as euthymic BD patients and thereby the SCZ and BD groups can be considered stable; differences in functional brain networks between SCZ and BD are therefore not related to a psychotic state, but may reflect trait differences between these groups.

Our findings are in-line with previous research showing decreased structural and functional connectivity in SCZ^12,14,21^ including an MST study that found decreased strength of structural connections but no differences in global MST topology in SCP and SCZ^15^. Together, these results are contradicting prior studies that reported increased functional connectivity^21,30^ and network topology differences in SCZ^20^. We suggest that less strict or no correction for possible connectivity strength effects in these studies may have led to less accurate estimations of network topology^17,21^. We found that medication effects cannot explain our results, as was already suggested by a prior study^31^. The fact that connectivity strength differences were found between SCZ and HC, while no topological differences were found, indicates that altered network topology is not a driving pathophysiological disease characteristic in SCZ. Rather, topographical characteristics of connectivity alterations, meaning the anatomical localization of network topology characteristics, may be specific to the disease.

Connectivity strength and MST topology did not differ between SCP and SCZ individuals. Previous work on cortical thickness and structural brain networks found that SCZ patients deviated more from controls than SCP individuals^13,16,32^ including a prior MST study using a partially overlapping dataset^15^. The lack of differences between SCP and SCZ individuals might be due to the small sample size of the SCP group. However, based on the small F values (<1.00) and effect sizes (~.01) of the post-hoc tests we do not expect to find a clinically relevant difference with a larger sample. Our global MST findings regarding BD are contradicting with research reporting no global differences in the structural connectome in (high risk for) BD^22,25^, similar disturbances in BD and SCZ^33–35^ or studies suggesting a spectrum with SCZ having decreased connectivity than HC and BD patients being intermediate^36^. Our results are also contrasting structural connectivity findings indicating that bipolar disorder is characterized by reduced global efficiency, impaired interhemispheric connectivity, and an unaffected rich club^26^. However, these studies are based on structural rather than functional MRI scans, and results relate to BD in general rather than to BD patients with a history of psychosis, which might explain the discrepancy to our results.

Regional MST alterations differ between SCZ and BD: In SCZ, frontal and occipital regions are more affected while in BD alterations are found in the temporal areas. Again, this might indicate that distinct neural correlates are related to psychosis in BD as compared to SCP and SCZ. Our findings replicated previous reports of decreased connectivity in frontal regions in SCZ and SCP^17,20,21,35^. Importantly, SCZ patients show increased connectivity in the right supramarginal gyrus, which might be related to auditory hallucinations since disrupted connectivity in language areas in SCP and SCZ might play a role in the experience of hallucinations^37^. This result is of interest in the light of findings of less lateralized language function in SCZ, with the right hemisphere showing increased language activity^38^. While the left hemisphere is language-dominant in HC, language function seems to be more bilateral in SCZ. Sommer et al.^38^ suggested that this might be due to increased language-related activity in the right hemisphere, which is consistent with our finding of increased connectivity in right-hemisphere language areas. Findings regarding BD are consistent with research reporting weaker structural connectivity in a subnetwork including the Rolandic operculum and neighboring fronto-temporal areas^25,26^.

We found no effect of antipsychotics or lithium on connectivity strength and MST topology as there was difference in MST measures neither between medication-naïve SCP individuals and SCZ patients, nor between BD patients that used antipsychotics or lithium and those that did not. This is in-line with findings that antipsychotic medication does not influence the blood-oxygenation-level-dependent signal in fMRI studies and is not significantly associated with connectivity measures^20,25,31^. However, it should be noted that several studies reported functional connectivity strength and network topology changes after treatment with antipsychotic medication, and that the SCP group may differ from medication naïve schizophrenia patients^39–43^. The effects of antidepressants and/or other mood stabilizers were not considered in this study but were not expected to affect findings^25^.

Strengths of this study are the direct comparisons of large, transdiagnostic samples with strict correction for motion effects and other confounders in network analysis including connectivity strength. A limitation of this study is that the groups differed in age, although our main findings were replicated in age-matched subgroups. Differences in age might suggest different disease stages, which may lead to different network organization^44^. Our SCZ cohort mainly consisted of chronic SCZ patients where network characteristics might contrast most with HC. Detailed data on disease duration were not available in our sample. Assessment of psychotic symptoms differed between groups, as a transdiagnostic instrument for the characterization of these symptoms such as the Questionnaire of Psychotic Experiences was not yet available during data collection^45^. However, we do not expect that other definitions of psychotic symptoms would lead to significant differences in our main findings. Regarding our methodology, we used the current state of art methodological pipeline but we cannot ensure that using other methods (e.g., using echo-planar fMRI, another atlas or connectivity measure, or using a seed-based or independent component analysis approach) might lead to different results.

In conclusion, functional networks may reflect a psychosis trait rather than state with different signatures for patients/healthy subjects in the schizophrenia spectrum compared to patients with bipolar disorder, which could not be explained by antipsychotic medication effects. No functional network alterations were found in the schizophrenia spectrum after strict correction for connectivity strength, suggesting that connectivity strength effects may have confounded previous functional network studies.

## Methods

### Participants

We included 97 patients with schizophrenia spectrum disorder, 136 patients with bipolar-I disorder, 35 individuals with subclinical psychotic experiences, and 219 healthy controls. Participants were recruited between 2006 and 2018 via the Dutch Bipolar Cohort (BD, HC^4,46^), The Outcome of Psychosis Fitness Therapy (SCZ, HC^47^), the Spectrum (SCP, SCZ, HC^48^), the Understanding Hallucinations (SCZ, HC; clinicaltrials.gov identifier NCT02460965), or the Simvastatin for recent onset psychosis studies (SCZ, HC^49^). All participants were above 18 years and had no diagnosis of alcohol or substance abuse disorders or somatic disorders (e.g., cardiovascular, neuromuscular, or endocrine disorders)^47,48^. The SCZ group had a diagnosis of schizophrenia, schizophreniform disorder, or psychosis not otherwise specified according to the DSM-IV^1^, had a lifetime history of hallucinations and/or delusions (as assessed by the Structured Clinical Interview for DSM-IV, SCID-I^50^, and the Comprehensive Assessment of Symptoms and History Interview, CASH^51^) and was stable on antipsychotic medication for at least 4 weeks before inclusion^47,49^. Subjects with a diagnosis of schizoaffective disorder (*N* = 12, 11%) were excluded to ensure contrast in the comparison between SCZ and BD. The BD group showed a lifetime history of hallucinations and/or delusions but was euthymic at the time of inclusion^52^. The SCP group had psychotic experiences at least once a month, had no diagnosis of an Axis I psychiatric disorder other than anxiety or depressive disorders in full remission, and was not using any psychiatric medication^32,48,53^. Two patients were using antidepressants in the SCP group, which is similar to the use of antidepressants in the general population; no other psychiatric medication was used in this group. The absence of a psychiatric diagnosis in the SCP group can be disputed since the criterion persistent hallucinations would be sufficient for a diagnosis of psychosis not otherwise specified (NOS). However, the general terms of the DSM state that a diagnosis should only be made if the symptoms and/or dysfunction bother the individual socially or occupationally, which was not the case in our study. The control group (recruited via all studies) included individuals with no current diagnosis but a history of depressive disorder (*N* = 17; 7.7%), ADHD (*N* = 1; 0.5%), PTSD (*N* = 1; 0.5%), adjustment disorder (*N* = 1; 0.5%), specific phobia (*N* = 2; 0.9%), mild alcohol use disorder (*N* = 3; 1.4%), conduct disorder (*N* = 1; 0.5%), or eating disorder (*N* = 1; 0.5%). Three controls (1.4%) were excluded due to the use of antidepressant medication. Characteristics of the participants are summarized in Table 2. Participants gave written informed consent and the studies were approved by the affiliated Institutional Review Board and conducted in accordance with the Declaration of Helsinki.

### Image acquisition and processing

Scans were acquired with a 3T Achieva Philips clinical MRI scanner equipped with an 8-channel SENSE head coil at the University Medical Center Utrecht (Philips, Best, The Netherlands). For anatomical reference, 3D high-resolution T1-weighted images were obtained with echo time [TE] = 4.6 ms, repetition time [TR] = 10 ms, flip angle = 8°, Field of View (FOV) = 240 mm/100%, voxel size = 0.75 × 0.75 × 0.80 mm, and reconstruction matrix = 200 × 320 × 320. For subjects recruited via the Spectrum study^48^, T1-weighted images were of lower resolution (160 contiguous sagittal slices, TE = 4.6 ms, TR = 10 ms, flip angle = 8°, FOV = 224 mm, voxel size = 1 × 1 × 1 mm). Apart from the T1-weighted images, all other scanning parameters were similar across studies so that it was not expected to impact our study. Resting-state functional MRI scans were acquired by combining a 3D PRESTO pulse sequence with parallel imaging (SENSE) in two directions leading to a fast functional brain coverage every 609 ms^54^. Parameters were set to 40 coronal slices, TE = 32.4 ms, TR = 21.75 ms, flip angle = 10°, FOV = 224 × 256 × 160, voxel size = 4 × 4 × 4 mm. Depending on the study, 600–1000 images were acquired. All resting-state scans were resized to the first 600 images (~6 min) and were checked for radiological abnormalities.

Images were processed using the FMRIB Software Library (FSL) version 5.0.4^55^. For preprocessing, FEAT’s default settings were used including skull stripping (BET), motion correction with MCFLIRT, spatial smoothing (5 mm kernel at full width at half maximum), and high-pass filtering (100-second cutoff). No global signal regression was performed as this could influence network topology analyses and group comparisons^56^. A systemic motion-related bias was prevented by excluding subjects whose relative root mean square displacement overall frames exceeded 0.2 mm or if 20 individual frames all exceeded the threshold of 0.25 mm^57^. ICA-AROMA was used to correct for in-scanner motion since it removes motion-related variance from the BOLD signal together with white matter and cerebral spinal fluid regressors^58,59^. From the initial 672 subjects analyzed, 185 subjects were excluded (HC *N* = 51; SCP *N* = 8; SCZ *N* = 54; BD *N* = 72) due to missing clinical data, processing errors, motion artifacts, or radiological exclusions resulting in 487 subjects included for further analysis (see Supplementary Table 1 for details).

### Connectivity analysis

Time series of each voxel across the brain were extracted and averaged across 264 functional regions according to the atlas described by Power et al.^60^. Subsequently, the brain network was reconstructed as a weighted graph from the resulting 264 time series. The cortical, subcortical, and cerebellar regions of the atlas described by Power and colleagues^60^ were used to define the graph’s nodes. Functional connections between nodes are represented as edges. Wavelet decompositions were applied to each of the time series, extracting wavelet coefficients in scale 4 (0.05–0.10 Hz)^61,62^. For wavelet filtering, the maximal overlap discrete wavelet transform (MODWT) method was used in the WMSTA toolbox (http://www.atmos.washington.edu/~wmsta/) in Matlab R2015b (The Mathworks, Inc). The edge weight or the strength of a connection was estimated by the wavelet coherence between the wavelet coefficients of two regions. The wavelet coherence was calculated using Welch’s overlapped averaged periodogram method^63^ for all possible node pairs, resulting in a weighted functional connectivity matrix of the size 264 × 264 for each subject.

### Minimum spanning tree analysis

Conventional graph theoretical measures might be influenced by the number of connections in the network and the connectivity strength^27–29,64^. For instance, lower connectivity strength is often found in patients as compared to control populations, which leads to lower values for network measures thereby confounding group comparisons of network topology^28^. To overcome these issues, the MST can be constructed, which is a subnetwork of the original graph that connects all nodes without forming loops and represents the network’s backbone^27,29^. The MST is relatively insensitive to differences in connectivity strength thereby enabling group comparisons of brain networks from different populations provided a similar number of nodes and a unique MST^29,65^.

Connectivity strength was defined as the mean of the edge weights in the MST matrix. MST diameter, kappa, and leaf fraction were calculated to characterize global network topology (see Fig. 1 and Table 1). Diameter measures the longest distance between the two most remote nodes in the network^27,29^, similar to the path length in conventional graph analysis. Kappa is a measure of diversity in nodal degree (kappa = <degree^2^>/<degree>). Leaf fraction quantifies the fraction of nodes in the whole network that have only one connecting edge and thereby is a measure of network integration, with higher leaf fraction indicating more integrated network topology. For regional network analyses, the degree and betweenness centrality were calculated (see Fig. 1): The degree states how many edges connect to a node^27,29^. Betweenness centrality measures how likely it is to pass a given node when connecting any two other nodes in the network. Together, these regional measures describe the nodal importance within the network.

### Statistical analysis

Statistical analyses were performed in IBM SPSS Statistics 25. Differences in subject characteristics were tested with one-way ANOVAs and Chi-Square tests. Normality of each outcome measure per group was assessed using the Shapiro–Wilk test and Q-Q plots. The Pearson correlation between motion (relative mean displacement) and connectivity strength was calculated to assure that motion did not confound the functional connectivity measures. As main analysis, we tested for group differences in global MST measures with ANCOVAs using the complete samples with age, sex, and years of education as covariates and Tukey LSD post-hoc tests. To correct for potential type I errors, the Benjamini–Hochberg procedure was used to correct for multiple testing. As sensitivity analysis, we manually matched the groups on age since age differences might bias differences in connectivity strength and brain network topology. Exploratory post-hoc ANCOVAs using the matched groups with age, sex, and education as covariates were performed to validate global MST group differences. Additionally, Matlab R2015b (The Mathworks, Inc) was used to investigate differences in local MST topology (i.e., differences in nodal degree and betweenness centrality) between the age-matched groups using permutation tests (10,000 permutations, Monte Carlo 2-sided test, Family Wise Error adjusted). To further check for medication effects, we repeated the ANCOVAs on global MST measures and the regional permutation tests comparing BD patients with (*N* = 66) and without antipsychotic medication (*N* = 65), and BD patients with (*N* = 85) and without (*N* = 51) lithium. The significance level for all statistical tests was set at *p* < 0.05.

### Reporting summary

Further information on research design is available in the Nature Research Reporting Summary linked to this article.

## Supplementary information

Supplemental material

reporting summary

## Data Availability

Data studied in this manuscript have been described previously^46–49^ (DOIs: 10.1017/S0033291715002299; 10.1016/j.euroneuro.2012.08.008; 10.1093/schbul/sbn130; 10.1016/j.bbacli.2015.06.007). Data can be accessed by contacting the authors of each respective paper.

## Code availability

Images were processed using the FMRIB Software Library (FSL) version 5.0.4. For wavelet filtering, the maximal overlap discrete wavelet transform (MODWT) method was used in the WMSTA toolbox (http://www.atmos.washington.edu/~wmsta/) in Matlab R2015b (The Mathworks, Inc). The MSTs were constructed in Matlab R2015b (The Mathworks, Inc) using personalized scripts, which are available from the authors upon request. The MSTs were visualized using the BrainNet Viewer (https://www.nitrc.org/projects/bnv/).
