# Supplementary material for: Functional brain networks in the schizophrenia spectrum and bipolar disorder with psychosis
Source: NPJ Schizophr. 2020 Sep 2;6:22. doi: 10.1038/s41537-020-00111-6 (PMC7468123; doi:10.1038/s41537-020-00111-6)
Supplement: Supplementary file 1 — Supplemental material [file 41537_2020_111_MOESM1_ESM.doc]

**Supplementary Materials**

**Subject Characteristics**

The reasons why we excluded participants from the current study can be found in Supplementary Table 1.

Descriptive statistics on age, sex and education for the matched groups can be found in Supplementary Tables 2-4.

Using the full sample, age and education showed the following differences: SCZ patients were significantly younger than HC, SCP, and BD individuals (*p* < .001), while BD subjects were significantly older than HC (*p* = .001). Regarding education, SCZ patients had significantly less years of education than HC (*p* < .001) and BD patients (*p* = .016).

Moreover, we calculated the Pearson’s correlation coefficient between the relative mean displacement (as calculated by the MCFLIRT function in FSL) and the connectivity measures to assure that motion has no influence on the functional connectivity analysis. The results are visualized in Supplementary Figure 1. The correlations between motion and connectivity measures were found to be close to zero indicating that the connectivity measures are not changing in relation to for instance increased movement during scanning. Therefore, it can be assumed that motion did not confound the functional connectivity and network topology findings.

**Matrix Visualizations**

The mean and median connectivity and the MST matrices did not differ between the groups.

**Group Comparisons**

The post-hoc results of the group comparisons of MST measures are summarized in Supplementary Table 5.

**Sensitivity analysis**

The results of the sensitivity analysis are summarized in the Supplementary Table 6. Sex had a significant effect as covariate on connectivity strength in the comparison between SCZ and HC (*F*(1, 189) = 5.536, *p* = .020, *ƞ^2^* = .028), with males having higher values than females, and education had a significant positive effect in the comparison between BD and SCZ (*F*(1, 117) = 4.968, *p* = .042, *ƞ^2^* = .041). When comparing BD and HC, age (*F*(1, 267) = 4.174, *p* = .042, *ƞ^2^* = .015) and sex (*F*(1, 267) = 4.146, *p* = .043, *ƞ^2^* = .015) had significant effects as covariates on the kappa scores, with higher kappa scores in males and in subjects with older age. Age also had a significant positive effect as covariate on the leaf fraction scores in the comparisons between BD and HC (*F*(1, 267) = 11.438, *p* = .001, *ƞ^2^* = .041) and between BD and SCP (*F*(1, 100) = 7.981, *p* = .006, *ƞ^2^* = .074).

We replicated the group comparisons for kappa scores with non-parametric tests due to the non-normal distribution of kappa scores. The results match our findings with parametric tests: Kappa scores did significantly differ between the groups (*H*(3) = 8.546, *p* = .036). Detailed post-hoc comparison results of Mann-Whitney U tests can be found in Supplementary Table 7.

**Regional Network Analysis**

Regional differences between networks of psychosis groups and the HC network are summarized in Supplementary Table 8. Permutation tests were used to investigate regional differences in betweenness centrality between the groups. The results regarding betweenness centrality are visualized in Supplementary Figure 2. Differences in betweenness centrality (significant at *p* = .05 after correction for multiple comparisons) showed the following pattern in comparison to HC: The bilateral frontal regions have a lower betweenness centrality in SCP, SCZ, and BD compared to HC. In addition, left occipital regions have a lower betweenness centrality in SCP and bilateral temporal regions have a lower betweenness centrality in BD relative to HC. The left anterior cingulate cortex has a higher betweenness centrality in BD compared to HC.

Regional differences between psychosis groups are summarized in Supplementary Table 9 and visualized in Supplementary Figures 3 (degree) and 4 (betweenness centrality). Permutation tests were used to investigate regional differences in MSTs between the groups. Differences in nodal degree (significant at *p* = .05 after correction for multiple comparisons) showed the following pattern: Right frontal and left occipital regions have a lower degree in BD compared to SCP, while bilateral temporal regions have a higher degree. In addition, bilateral (fronto-) temporal regions have a lower degree in BD relative to SCZ. Finally, the left angular gyrus has a lower degree in SCZ compared to SCP, while right occipital regions have a higher degree. Differences in betweenness centrality (significant at *p* = .05 after correction for multiple comparisons) showed the following pattern: Bilateral occipital regions have a lower betweenness centrality in BD compared to SCP and SCZ, while bilateral fronto-temporal and temporo-occipital regions have a higher betweenness centrality. The left angular gyrus has a lower betweenness centrality in SCZ relative to SCP, while bilateral frontal and right temporal regions have a higher betweenness centrality.

**Medication Effects**

Regional differences between BD patients that used antipsychotics and those that did not are summarized in Supplementary Table 10 and visualized in Supplementary Figure 5. The right calcarine sulcus has a higher degree and betweenness centrality in BD patients that used antipsychotics than in those that did not. The left triangular inferior frontal gyrus and precuneus have a lower degree. In addition, the left and right supplementary motor areas and middle temporal gyri, the left parahippocampal gyrus and fusiform area, and the right superior and inferior temporal gyri, angular gyrus, postcentral gyrus, insula, and putamen have a lower betweenness centrality in BD patients that used antipsychotics than in those that did not.

**Supplementary Figures**

**
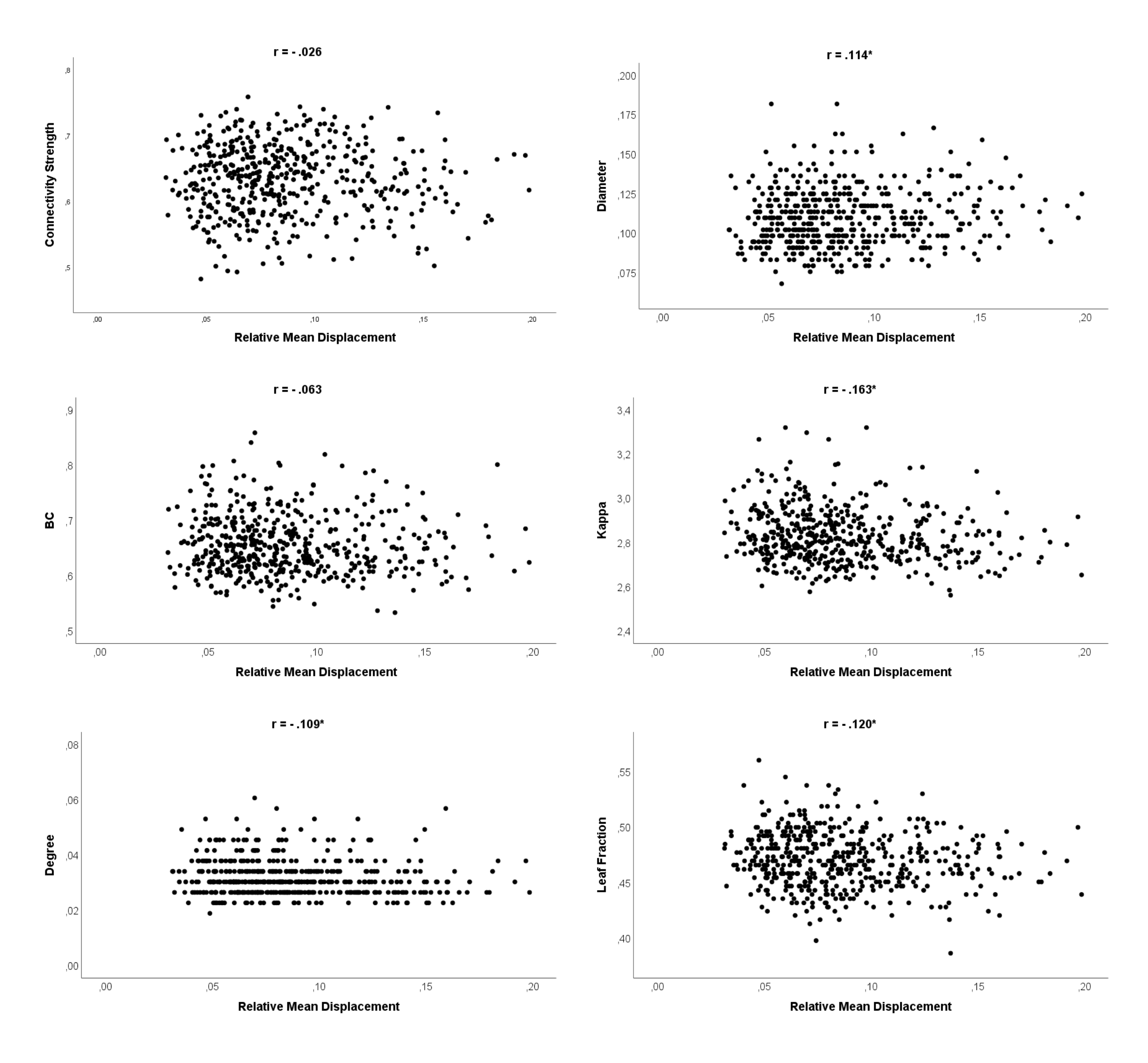
**

*Supplementary Figure 1.* Correlations between motion (relative mean displacement) and the MST measures.

Pearson’s correlations that are significant at *p* = .05 are marked with an asterisk (*).

*
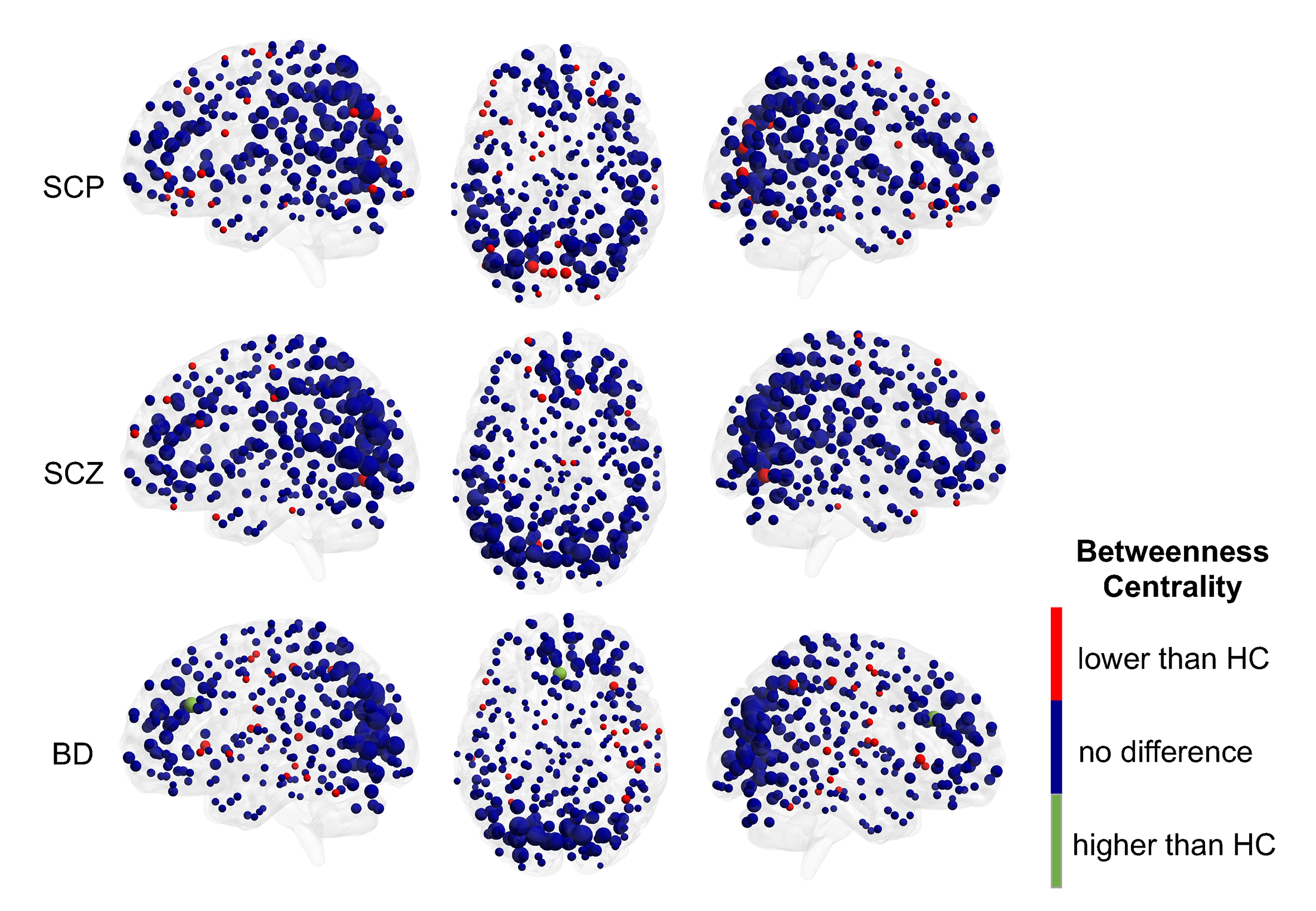
*

*Supplementary Figure 2.* Visualization of regional differences in betweenness centrality between the networks of psychosis groups and the healthy control network.

Healthy controls were age-matched. The size of the nodes corresponds to the betweenness centrality of the nodes. Blue nodes mark regions that do not differ compared to healthy controls, while red nodes mark regions with a significantly lower betweenness centrality and green nodes mark regions with a significantly higher betweenness centrality compared to controls. Abbreviations: HC: healthy controls, SCP: sub-clinical psychosis, SCZ: schizophrenia spectrum disorder, BD: bipolar disorder with psychosis

*
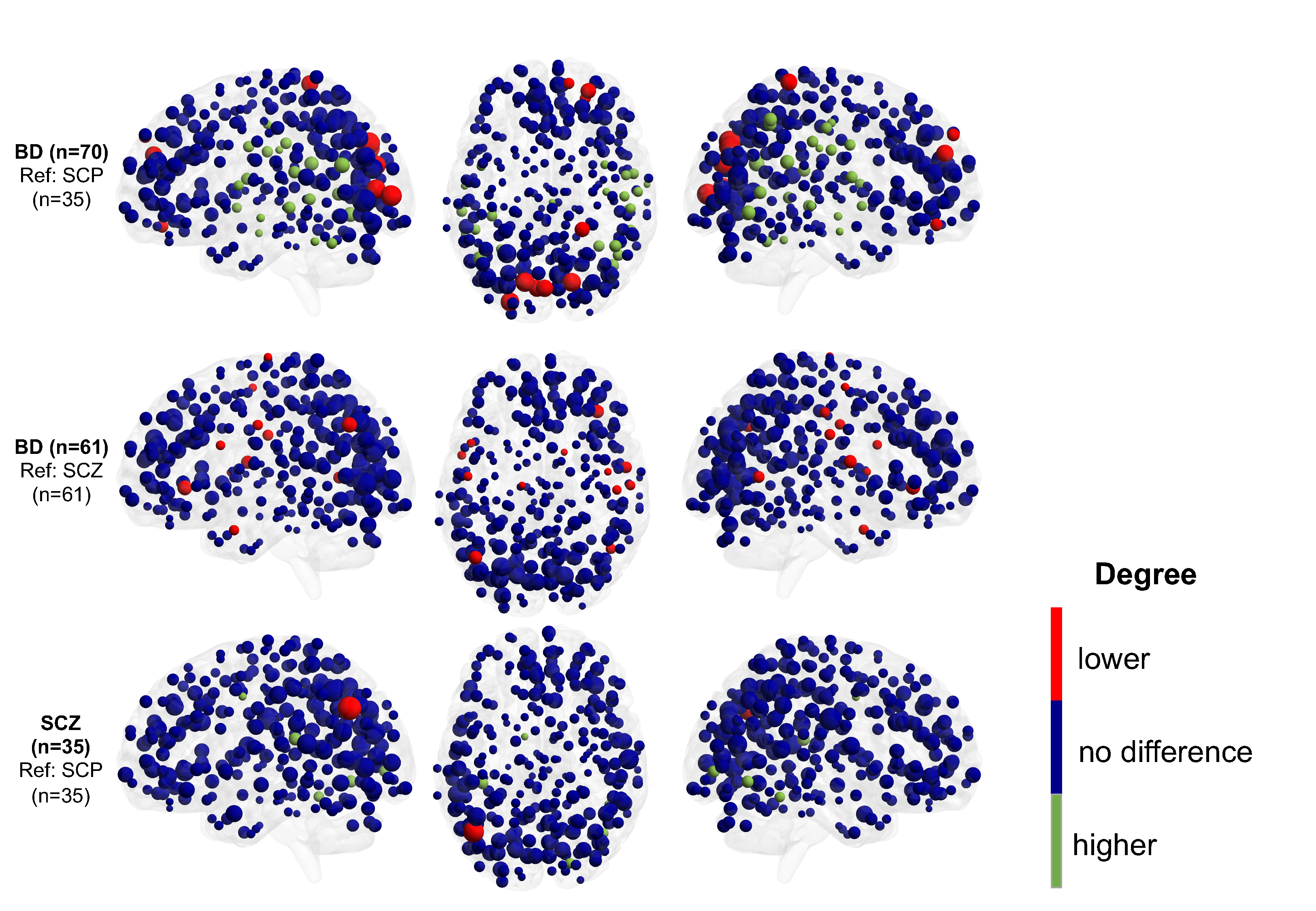
*

*Supplementary Figure 3.* Visualization of regional differences in degree between the networks of psychosis groups.

Psychosis groups were age-matched. The size of the nodes corresponds to the degree of the nodes. Blue nodes mark regions that do not differ compared to the reference, while red nodes mark regions with a significantly lower degree and green nodes mark regions with a significantly higher degree compared to the reference. Abbreviations: HC: healthy controls, SCP: sub-clinical psychosis, SCZ: schizophrenia spectrum disorder, BD: bipolar disorder with psychosis

*
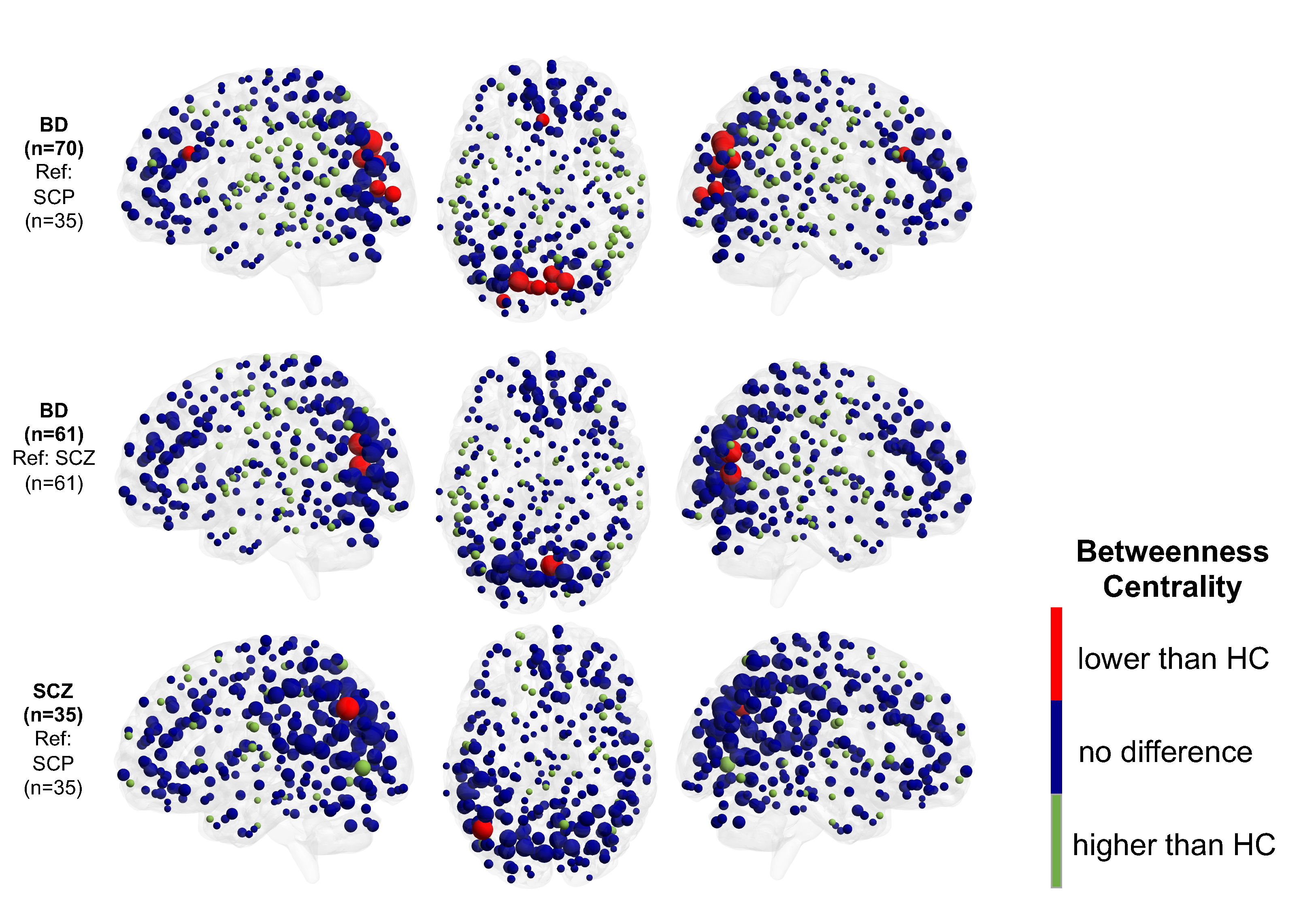
*

*Supplementary Figure 4.* Visualization of regional differences in betweenness centrality (BC) between the networks of psychosis groups.

Psychosis groups were age-matched. The size of the nodes corresponds to the BC of the nodes. Blue nodes mark regions that do not differ compared to the reference, while red nodes mark regions with a significantly lower BC and green nodes mark regions with a significantly higher BC compared to the reference. Abbreviations: HC: healthy controls, SCP: sub-clinical psychosis, SCZ: schizophrenia spectrum disorder, BD: bipolar disorder with psychosis

*
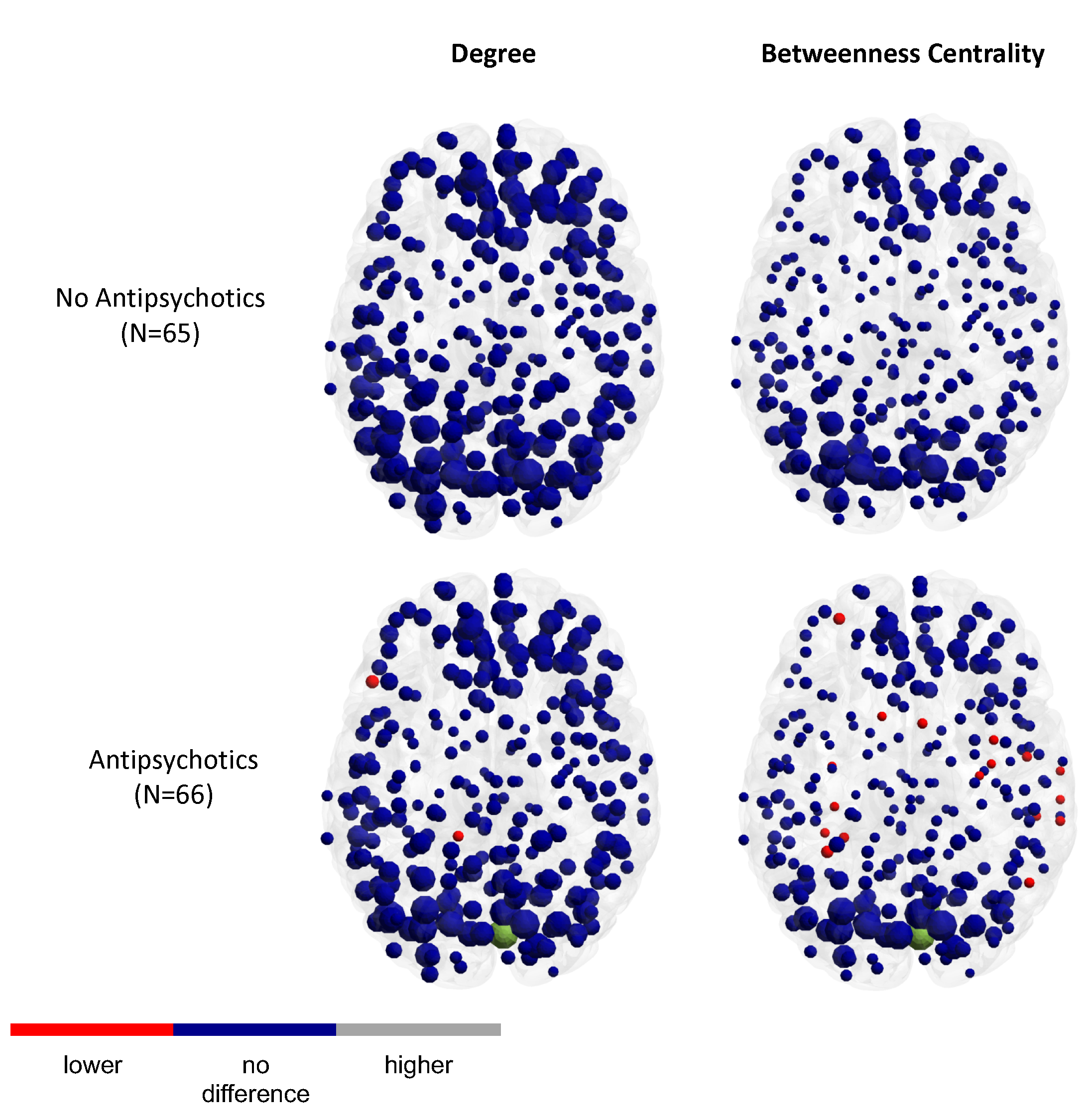
*

*Supplementary Figure 5.* Visualization of regional differences between the networks of bipolar disorder patients that used antipsychotics and those that did not.

The size of the nodes corresponds to the degree and betweenness centrality, respectively. Blue nodes mark regions that do not differ between groups, while red nodes mark regions with a significantly lower degree or betweenness centrality and green nodes mark regions with a significantly higher degree or betweenness centrality than the comparison group.

**Supplementary Tables**

Supplementary Table 1

Reasons for Exclusions of Subjects

| Reason for Exclusion | SCP | SCZ | BD | HC |
| --- | --- | --- | --- | --- |
| Missing Data | 1 | 11 | 0 | 5 |
| Processing Errors | 1 | 13 | 38 | 36 |
| Motion Artefacts | 5 | 29 | 29 | 8 |
| Radiological Exclusions | 1 | 1 | 5 | 2 |
| Total | 8 | 54 | 72 | 51 |
| Total Sample | N = 672 | | | |
| Total Excluded | N = 185 | | | |
| Total Included | N = 487 | | | |

Abbreviations: SCP sub-clinical psychosis, SCZ schizophrenia spectrum disorder, BD bipolar disorder with psychosis, HC healthy controls

Supplementary Table 2

Age Descriptives for the Psychosis Groups and their Matched Groups

| Matched Groups | N | Mean | SD |
| --- | --- | --- | --- |
| SCP | 35 | 42.09 | 15.04 |
| HC | 70 | 42.16 | 14.77 |
| SCZ | 97 | 31.46 | 10.56 |
| HC | 97 | 31.79 | 10.50 |
| BD | 136 | 46.12 | 11.65 |
| HC | 136 | 46.04 | 11.58 |
| SCZ | 35 | 38.40 | 13.78 |
| SCP | 35 | 42.09 | 15.04 |
| BD | 70 | 43.36 | 13.30 |
| SCP | 35 | 42.09 | 15.04 |
| BD | 61 | 37.23 | 10.52 |
| SCZ | 61 | 35.93 | 10.51 |
| BD/AP | 66 | 44.14 | 11.21 |
| BD/nAP | 65 | 47.71 | 12.18 |

Abbreviations: HC: healthy controls, SCP: sub-clinical psychosis, SCZ: schizophrenia spectrum disorder, BD: bipolar disorder with psychosis, BD/AP: bipolar disorder with antipsychotic use, BD/nAP: bipolar disorder without antipsychotic use

Supplementary Table 3

Sex Descriptives for the Psychosis Groups and their Matched Groups

| Matched Groups | N | % female | % male |
| --- | --- | --- | --- |
| SCP | 35 | 74.3 | 25.7 |
| HC | 70 | 64.3 | 35.7 |
| SCZ | 97 | 32.0 | 68.0 |
| HC | 97 | 42.3 | 57.7 |
| BD | 136 | 50.7 | 49.3 |
| HC | 136 | 47.1 | 52.9 |
| SCZ | 35 | 40.0 | 60.0 |
| SCP | 35 | 74.3 | 25.7 |
| BD | 70 | 47.1 | 52.9 |
| SCP | 35 | 74.3 | 25.7 |
| BD | 61 | 52.5 | 47.5 |
| SCZ | 61 | 34.4 | 65.6 |

Abbreviations: HC: healthy controls, SCP: sub-clinical psychosis, SCZ: schizophrenia spectrum disorder, BD: bipolar disorder with psychosis, BD/AP: bipolar disorder with antipsychotic use, BD/nAP: bipolar disorder without antipsychotic use

Supplementary Table 4

Education Descriptives for the Psychosis Groups and their Matched Groups

| Matched Groups | N | Mean | SD |
| --- | --- | --- | --- |
| SCP | 35 | 13.63 | 1.85 |
| HC | 70 | 13.57 | 2.73 |
| SCZ | 97 | 12.35 | 3.33 |
| HC | 97 | 14.29 | 2.59 |
| BD | 136 | 13.50 | 2.90 |
| HC | 136 | 13.80 | 2.80 |
| SCZ | 35 | 12.16 | 2.95 |
| SCP | 35 | 13.63 | 1.85 |
| BD | 70 | 13.29 | 3.10 |
| SCP | 35 | 13.63 | 1.85 |
| BD | 61 | 13.77 | 2.68 |
| SCZ | 61 | 12.60 | 3.20 |

Abbreviations: HC: healthy controls, SCP: sub-clinical psychosis, SCZ: schizophrenia spectrum disorder, BD: bipolar disorder with psychosis, BD/AP: bipolar disorder with antipsychotic use, BD/nAP: bipolar disorder without antipsychotic use

Supplementary Table 5

Post-hoc Results of Minimum Spanning Tree Group Analyses

|  | Groups | | Absolute Mean Difference (SE) | p |
| --- | --- | --- | --- | --- |
| Connectivity Strength |  |  |  |  |
|  | HC | SCP | .021 (.009) | .029* |
|  | HC | SCZ | .023 (.007) | .001* |
|  | HC | BD | -.001 (.006) | .793 |
|  | SCP | SCZ | .002 (.011) | .836 |
|  | SCP | BD | -.022 (.010) | .024* |
|  | SCZ | BD | -.024 (.008) | .001* |
| Kappa |  |  |  |  |
|  | HC | SCP | -.026 (.022) | .233 |
|  | HC | SCZ | .005 (.016) | .728 |
|  | HC | BD | .039 (.013) | .004* |
|  | SCP | SCZ | .032 (.025) | .197 |
|  | SCP | BD | .065 (.023) | .005* |
|  | SCZ | BD | .033 (.017) | .058 |
| Leaf Fraction |  |  |  |  |
|  | HC | SCP | -.006 (.004) | .183 |
|  | HC | SCZ | .001 (.003) | .787 |
|  | HC | BD | .007 (.003) | .011* |
|  | SCP | SCZ | .007 (.005) | .174 |
|  | SCP | BD | .013 (.005) | .006* |
|  | SCZ | BD | .006 (.004) | .092 |

Group differences were tested with Tukey LSD post-hoc tests. Significant differences at p = .05 are marked with an asterisk (*). Abbreviations: HC: healthy controls, SCP: sub-clinical psychosis, SCZ: schizophrenia spectrum disorder, BD: bipolar disorder with psychosis

Supplementary Table 6

Post-hoc Results of Minimum Spanning Tree Group Analyses with Matched Groups

|  | **Groups** | | **Absolute Mean Difference (SE)** | ***df*** | ***F*** | ***p*** | ***Ƞ^2^*** |
| --- | --- | --- | --- | --- | --- | --- | --- |
| **Connectivity Strength** |  |  |  |  |  |  |  |
|  | **HC** | **SCP** | .019 (.011) | 1, 95 | 3.213 | .076 | .031 |
|  |  | Age |  | 1, 95 | 2.074 | .153 | .020 |
|  |  | Sex |  | 1, 95 | .080 | .778 | .001 |
|  |  | Education |  | 1, 95 | .504 | .551 | .005 |
|  | **HC** | **SCZ** | .020 (.008) | 1, 189 | 6.956 | .010* | .035 |
|  |  | Age |  | 1, 189 | .996 | .323 | .005 |
|  |  | Sex |  | 1, 189 | 5.536 | .020* | .028 |
|  |  | Education |  | 1, 189 | 3.889 | .058 | .020 |
|  | **HC** | **BD** | -.001 (.006) | 1, 267 | .045 | .833 | < .001 |
|  |  | Age |  | 1, 267 | .227 | .636 | .001 |
|  |  | Sex |  | 1, 267 | 1.818 | .178 | .007 |
|  |  | Education |  | 1, 267 | 1.780 | .203 | .007 |
|  | **SCP** | **SCZ** | .009 (.015) | 1, 65 | .409 | .546 | .006 |
|  |  | Age |  | 1, 65 | 1.632 | .206 | .024 |
|  |  | Sex |  | 1, 65 | .067 | .813 | .001 |
|  |  | Education |  | 1, 65 | .350 | .646 | .005 |
|  | **SCP** | **BD** | -.028 (.010) | 1, 100 | 7.123 | .009* | .066 |
|  |  | Age |  | 1, 100 | .158 | .691 | .002 |
|  |  | Sex |  | 1, 100 | .114 | .737 | .001 |
|  |  | Education |  | 1, 100 | .330 | .567 | .003 |
|  | **SCZ** | **BD** | -.027 (.010) | 1, 117 | 8.143 | .006* | .065 |
|  |  | Age |  | 1, 117 | .521 | .474 | .004 |
|  |  | Sex |  | 1, 117 | .583 | .448 | .005 |
|  |  | Education |  | 1, 117 | 4.968 | .042* | .041 |
| **Kappa** |  |  |  |  |  |  |  |
|  | **HC** | **SCP** | -.031 (.026) | 1, 95 | 1.392 | .241 | .014 |
|  |  | Age |  | 1, 95 | .397 | .530 | .004 |
|  |  | Sex |  | 1, 95 | .002 | .964 | < .001 |
|  |  | Education |  | 1, 95 | .392 | .563 | .004 |
|  | **HC** | **SCZ** | .008 (.019) | 1, 189 | .199 | .681 | .001 |
|  |  | Age |  | 1, 189 | .473 | .494 | .003 |
|  |  | Sex |  | 1, 189 | .420 | .519 | .002 |
|  |  | Education |  | 1, 189 | .277 | .653 | .002 |
|  | **HC** | **BD** | .040 (.014) | 1, 267 | 7.805 | .006* | .028 |
|  |  | Age |  | 1, 267 | 4.174 | .042* | .015 |
|  |  | Sex |  | 1, 267 | 4.146 | .043* | .015 |
|  |  | Education |  | 1, 267 | .923 | .366 | .003 |
|  | **SCP** | **SCZ** | .030 (.037) | 1, 65 | .649 | .427 | .010 |
|  |  | Age |  | 1, 65 | .006 | .939 | < .001 |
|  |  | Sex |  | 1, 65 | .489 | .488 | .007 |
|  |  | Education |  | 1, 65 | .391 | .560 | .006 |
|  | **SCP** | **BD** | .069 (.025) | 1, 100 | 7.779 | .006* | .072 |
|  |  | Age |  | 1, 100 | 3.155 | .079 | .031 |
|  |  | Sex |  | 1, 100 | .209 | .649 | .002 |
|  |  | Education |  | 1, 100 | .291 | .591 | .003 |
|  | **SCZ** | **BD** | .028 (.022) | 1, 117 | 1.609 | .207 | .014 |
|  |  | Age |  | 1, 117 | 5.242 | .024* | .043 |
|  |  | Sex |  | 1, 117 | .144 | .705 | .001 |
|  |  | Education |  | 1, 117 | .121 | .743 | .001 |
| **Leaf Fraction** |  |  |  |  |  |  |  |
|  | **HC** | **SCP** | -.004 (.005) | 1, 95 | .798 | .374 | .008 |
|  |  | Age |  | 1, 95 | 1.153 | .286 | .011 |
|  |  | Sex |  | 1, 95 | .026 | .875 | < .001 |
|  |  | Education |  | 1, 95 | 2.000 | .178 | .019 |
|  | **HC** | **SCZ** | .001 (.004) | 1, 189 | .158 | .697 | .001 |
|  |  | Age |  | 1, 189 | 1.189 | .277 | .006 |
|  |  | Sex |  | 1, 189 | .740 | .391 | .004 |
|  |  | Education |  | 1, 189 | .236 | .677 | .001 |
|  | **HC** | **BD** | .007 (.003) | 1, 267 | 5.163 | .024* | .019 |
|  |  | Age |  | 1, 267 | 11.438 | .001* | .041 |
|  |  | Sex |  | 1, 267 | 2.431 | .120 | .009 |
|  |  | Education |  | 1, 267 | .319 | .611 | .001 |
|  | **SCP** | **SCZ** | .007 (.007) | 1, 65 | .850 | .366 | .013 |
|  |  | Age |  | 1, 65 | < .001 | .990 | < .001 |
|  |  | Sex |  | 1, 65 | .024 | .886 | .001 |
|  |  | Education |  | 1, 65 | .213 | .727 | .003 |
|  | **SCP** | **BD** | .014 (.005) | 1, 100 | 7.701 | .007* | .071 |
|  |  | Age |  | 1, 100 | 7.981 | .006* | .074 |
|  |  | Sex |  | 1, 100 | .007 | .932 | < .001 |
|  |  | Education |  | 1, 100 | .502 | .480 | .005 |
|  | **SCZ** | **BD** | .010 (.004) | 1, 117 | 4.801 | .030* | .039 |
|  |  | Age |  | 1, 117 | 3.142 | .079 | .026 |
|  |  | Sex |  | 1, 117 | .337 | .563 | .003 |
|  |  | Education |  | 1, 117 | .038 | .872 | .001 |

Group differences were tested with ANCOVAs with age, sex, and education as covariates using age-matched groups. Significant differences at *p* = .05 are marked with an asterisk (*). Abbreviations: HC: healthy controls, SCP: sub-clinical psychosis, SCZ: schizophrenia spectrum disorder, BD: bipolar disorder with psychosis

Supplementary Table 7

Post-hoc Results of Non-Parametric Group Comparisons of Kappa Scores

| Groups | | U | p |
| --- | --- | --- | --- |
| HC | SCP | 3554.00 | .490 |
| HC | SCZ | 9678.00 | .208 |
| HC | BD | 12,385.00 | .008* |
| SCP | SCZ | 1479.00 | .260 |
| SCP | BD | 1881.50 | .056 |
| SCZ | BD | 6093.50 | .322 |

Group differences were tested with Mann-Whitney U tests. Significant differences at p = .05 are marked with an asterisk (*). Abbreviations: HC: healthy controls, SCP: sub-clinical psychosis, SCZ: schizophrenia spectrum disorder, BD: bipolar disorder with psychosis

Supplementary Table 8

Regional Differences in Degree and Betweenness Centrality Between Psychosis Groups and Matched Control Groups

| Psychosis Group | Degree | | Betweenness Centrality | |
| --- | --- | --- | --- | --- |
|  | Higher | Lower | Higher | Lower |
| SCP |  |  |  |  |
| Bilateral |  |  |  | Lingual Gyrus  Frontal Gyrus  Orbitofrontal Cortex  Calcarine Sulcus  Vermis |
| Left |  | Angular Gyrus  Cuneus |  | Angular Gyrus  Midcingulate Area  Temporal Pole  SMA  Occipital Gyrus  Cuneus |
| Right |  | Gyrus Rectus |  | Gyrus Rectus  Temporal Gyrus |
| SCZ |  |  |  |  |
| Bilateral |  |  |  | Frontal Gyrus |
| Left |  | Frontal Gyrus  ACC  Cuneus |  | Fusiform Area  Lingual Gyrus |
| Right | Supramarginal Gyrus | SMA |  | Gyrus Rectus  SMA  Temporal Pole |
| BD |  |  |  |  |
| Bilateral |  |  |  | Temporal Gyrus |
| Left | Occipital Gyrus  ACC |  | ACC | Midcingulate Area  Temporal Pole  Fusiform Area  Frontal Gyrus  Cerebellum |
| Right | Frontal Gyrus  Precuneus | Precentral Gyrus  Postcentral Gyrus  Insula  Rolandic Operculum  Temporal Gyrus |  | Precentral Gyrus  Postcentral Gyrus  Insula  Rolandic Operculum  Parietal Gyrus |

Abbreviations: SMA supplementary motor area, ACC anterior cingulate cortex, SCP sub-clinical psychosis, SCZ schizophrenia spectrum disorder, BD bipolar disorder with psychosis

Supplementary Table 9

Regional Differences in Degree and Betweenness Centrality Between Matched Psychosis Groups

| Psychosis Group | Degree | | Betweenness Centrality | |
| --- | --- | --- | --- | --- |
|  | Higher | Lower | Higher | Lower |
| BD  (reference: SCP) |  |  |  |  |
| Bilateral | Rolandic Operculum  Temporal Gyrus | Cuneus | Rolandic Operculum  Temporal Gyrus  Precentral Gyrus  Postcentral Gyrus  Temporal Pole  Supramarginal Gyrus  Angular Gyrus  Frontal Gyrus  Midcingulate Area  Precuneus | Cuneus  Calcarine Sulcus |
| Left | Cerebellum | Occipital Gyrus Calcarine Sulcus | Cerebellum  Fusiform Area  PCC  PHG  Thalamus | Occipital Gyrus  ACC |
| Right | Parietal Gyrus  Precentral Gyrus  Postcentral Gyrus  Supramarginal Gyrus  Angular Gyrus  Midcingulate Area | Frontal Gyrus  Orbitofrontal Cortex | Parietal Gyrus  SMA  Insula  Occipital Gyrus  Lingual Gyrus |  |
| BD  (reference: SCZ) |  |  |  |  |
| Bilateral | Postcentral Gyrus  Temporal Gyrus |  | Postcentral Gyrus  Temporal Gyrus  SMA  Precentral Gyrus  Angular Gyrus  Putamen |  |
| Left | Paracentral Lobule  Frontal Gyrus  Angular Gyrus |  | Paracentral Lobule  Frontal Gyrus  Parietal Gyrus  Temporal Pole  PCC  Cerebellum |  |
| Right | Rolandic Operculum  Orbitofrontal Cortex Precentral Gyrus |  | Rolandic Operculum  Orbitofrontal Cortex  Insula  Occipital Gyrus  Lingual Gyrus | Calcarine Sulcus  Cuneus |
| SCZ  (reference: SCP) |  |  |  |  |
| Bilateral |  |  | Postcentral Gyrus  Frontal Gyrus  Thalamus  Precuneus |  |
| Left | Midcingulate Area  Rolandic Operculum | Angular Gyrus | Midcingulate Area  Rolandic Operculum  Orbitofrontal Cortex  Occipital Gyrus | Angular Gyrus |
| Right | Temporal Gyrus Lingual Gyrus |  | Temporal Gyrus  PHG  Paracentral Lobule  Precentral Gyrus  Temporal Pole  Insula  Putamen  Fusiform Area |  |

Abbreviations: SMA supplementary motor area, PHG parahippocampal gyrus, ACC anterior cingulate cortex, PCC posterior cingulate cortex, SCP sub-clinical psychosis, SCZ schizophrenia spectrum disorder, BD bipolar disorder with psychosis

Supplementary Table 10

Regional Differences in Degree and Betweenness Centrality Between Bipolar Disorder Patients With and Without Antipsychotic Use

|  | Bipolar Disorder with Antipsychotics | |
| --- | --- | --- |
|  | Left | Right |
| Degree |  |  |
| Higher |  | Calcarine Sulcus |
| Lower | Triangular inferior frontal gyrus,  Precuneus |  |
| Betweenness Centrality |  |  |
| Higher |  | Calcarine Sulcus |
| Lower | Supplementary motor area,  Middle temporal gyrus,  Parahippocampal gyrus,  Fusiform area | Supplementary motor area,  Middle temporal gyrus,  Superior and inferior temporal gyri,  Angular gyrus,  Postcentral gyrus,  Insula,  Putamen |
