# Supplementary material for: Functional brain networks in the schizophrenia spectrum and bipolar disorder with psychosis
Source: NPJ Schizophr. 2020 Sep 2;6:22. doi: 10.1038/s41537-020-00111-6 (PMC7468123; doi:10.1038/s41537-020-00111-6)
Supplement: Supplementary file 2 — reporting summary [file 41537_2020_111_MOESM2_ESM.pdf]

nature research

Corresponding author(s): *Double-blind peer review submissions: write DBPR and your manuscript number here instead of author names.*

Last updated by author(s): *YYYY-MM-DD*

Reporting Summary

Nature Research wishes to improve the reproducibility of the work that we publish. This form provides structure for consistency and transparency in reporting. For further information on Nature Research policies, see Authors & Referees and the Editorial Policy Checklist.

Statistics

For all statistical analyses, confirm that the following items are present in the figure legend, table legend, main text, or Methods section.

n/a	Confirmed	
☐	☒	The exact sample size (n) for each experimental group/condition, given as a discrete number and unit of measurement
☐	☒	A statement on whether measurements were taken from distinct samples or whether the same sample was measured repeatedly
☐	☒	The statistical test(s) used AND whether they are one- or two-sided<br>*Only common tests should be described solely by name; describe more complex techniques in the Methods section.*
☐	☒	A description of all covariates tested
☐	☒	A description of any assumptions or corrections, such as tests of normality and adjustment for multiple comparisons
☐	☒	A full description of the statistical parameters including central tendency (e.g. means) or other basic estimates (e.g. regression coefficient) AND variation (e.g. standard deviation) or associated estimates of uncertainty (e.g. confidence intervals)
☐	☒	For null hypothesis testing, the test statistic (e.g. F, t, r) with confidence intervals, effect sizes, degrees of freedom and P value noted<br>*Give P values as exact values whenever suitable.*
☒	☐	For Bayesian analysis, information on the choice of priors and Markov chain Monte Carlo settings
☒	☐	For hierarchical and complex designs, identification of the appropriate level for tests and full reporting of outcomes
☐	☒	Estimates of effect sizes (e.g. Cohen's d, Pearson's r), indicating how they were calculated

Our web collection on statistics for biologists contains articles on many of the points above.

Software and code

Policy information about availability of computer code

Data collection	N.A.
Data analysis	Images were processed using the FMRIB Software Library (FSL) version 5.0.4 [40]. For preprocessing, FEAT's default settings were used including skull stripping (BET), motion correction with MCFLIRT. For wavelet filtering, the maximal overlap discrete wavelet transform (MODWT) method was used in the WMSTA toolbox (http://www.atmos.washington.edu/~wmsta/) in Matlab R2015b (The Mathworks, Inc). The MSTs were constructed based on individuals as well as the group median using Kruskal's algorithm [51] in Matlab R2015b (The Mathworks, Inc). The MSTs were visualized using the BrainNet Viewer [52] (https://www.nitrc.org/projects/bnv/).

For manuscripts utilizing custom algorithms or software that are central to the research but not yet described in published literature, software must be made available to editors/reviewers. We strongly encourage code deposition in a community repository (e.g. GitHub). See the Nature Research guidelines for submitting code & software for further information.

Data

Policy information about availability of data

All manuscripts must include a data availability statement. This statement should provide the following information, where applicable:

- Accession codes, unique identifiers, or web links for publicly available datasets
- A list of figures that have associated raw data
- A description of any restrictions on data availability

No consent was given for making data publically available by participants.

Field-specific reporting

Please select the one below that is the best fit for your research. If you are not sure, read the appropriate sections before making your selection.

☒ Life sciences ☐ Behavioural & social sciences ☐ Ecological, evolutionary & environmental sciences

For a reference copy of the document with all sections, see nature.com/documents/nr-reporting-summary-flat.pdf

Life sciences study design

All studies must disclose on these points even when the disclosure is negative.

Sample size	Sample sizes were sufficient for case-control comparisons (Friston, 10 Ironic rules for non-statistical reviewers, NeuroImage).
Data exclusions	From the initial 672 subjects analyzed, 185 subjects were excluded (HC N = 51; SCP N = 8; SCZ N = 54; BD N = 72) due to missing clinical data, processing errors, motion artifacts, or radiological exclusions resulting in 487 subjects included for further analysis (see Supplementary Table 1 for details).
Replication	replication data were not available to the authors.
Randomization	N.A., case control study.
Blinding	N.A., case control study.

Reporting for specific materials, systems and methods

We require information from authors about some types of materials, experimental systems and methods used in many studies. Here, indicate whether each material, system or method listed is relevant to your study. If you are not sure if a list item applies to your research, read the appropriate section before selecting a response.

Materials & experimental systems

n/a	Involved in the study	
☒	☐	Antibodies
☒	☐	Eukaryotic cell lines
☒	☐	Palaeontology
☒	☐	Animals and other organisms
☐	☒	Human research participants
☐	☒	Clinical data

Methods

n/a	Involved in the study	
☒	☐	ChIP-seq
☒	☐	Flow cytometry
☐	☒	MRI-based neuroimaging

Human research participants

Policy information about studies involving human research participants

Population characteristics	We included 97 patients with schizophrenia spectrum disorder, 136 patients with bipolar-I disorder, 35 individuals with sub-clinical psychotic experiences, and 219 healthy controls. All participants were above 18 years and had no diagnosis of alcohol or substance abuse disorders or somatic disorders (e.g., cardiovascular, neuromuscular, or endocrine disorders) [31, 32]. The SCZ group had a diagnosis of schizophrenia, schizophreniform disorder, or psychosis not otherwise specified according to the DSM-IV [1], had a lifetime history of hallucinations and/or delusions (as assessed by the Structured Clinical Interview for DSM-IV, SCID-I [34], and the Comprehensive Assessment of Symptoms and History Interview, CASH [35]) and was stable on antipsychotic medication for at least four weeks before inclusion [31, 33]. Subjects with a diagnosis of schizoaffective disorder (N = 12, 11%) were excluded to ensure contrast in the comparison between SCZ and BD. The BD group showed a lifetime history of hallucinations and/or delusions but was euthymic at the time of inclusion [36]. The SCP group had psychotic experiences at least once a month, had no diagnosis of an Axis I psychiatric disorder other than anxiety or depressive disorders in full remission, and was not using any psychiatric medication [32, 37, 38]. Two patients were using antidepressants in the SCP group, which is similar to the use of antidepressants in the general population; no other psychiatric medication was used in this group. The absence of a psychiatric diagnosis in the SCP group can be disputed since the criterion persistent hallucinations would be sufficient for a diagnosis of psychosis not otherwise specified (NOS). However, the general terms of the DSM state that a diagnosis should only be made if the symptoms and/or dysfunction bother the individual socially or occupationally, which was not the case in our study. The control group (recruited via all studies) included individuals with no current diagnosis but a history of depressive disorder (N = 17; 7.7%), ADHD (N = 1; 0.5%), PTSD (N = 1; 0.5%), adjustment disorder (N = 1; 0.5%), specific phobia (N = 2; 0.9%), mild alcohol use disorder (N = 3; 1.4%), conduct disorder (N = 1; .5%), or eating disorder (N = 1; 0.5%). Three controls (1.4%) were excluded due to the use of antidepressant medication. Characteristics of the participants are summarized in Table 1.

Recruitment	Participants were recruited between 2006 and 2018 via the Dutch Bipolar Cohort (BD, HC; [4, 30]), The Outcome of Psychosis Fitness Therapy (SCZ, HC; [31]), the Spectrum (SCP, SCZ, HC; [32]), the Understanding Hallucinations (SCZ, HC; clinicaltrials.gov identifier NCT02460965), or the Simvastatin for recent onset psychosis studies (SCZ, HC; [33]).
Ethics oversight	Participants gave written informed consent and the studies were approved by the affiliated Institutional Review Board and conducted in accordance with the Declaration of Helsinki.

Note that full information on the approval of the study protocol must also be provided in the manuscript.

Clinical data

Policy information about clinical studies
All manuscripts should comply with the ICMJE guidelines for publication of clinical research and a completed CONSORT checklist must be included with all submissions.

Clinical trial registration	Participants were recruited between 2006 and 2018 via the Dutch Bipolar Cohort (BD, HC; [4, 30]), The Outcome of Psychosis Fitness Therapy (SCZ, HC; [31]), the Spectrum (SCP, SCZ, HC; [32]), the Understanding Hallucinations (SCZ, HC; clinicaltrials.gov identifier NCT02460965), or the Simvastatin for recent onset psychosis studies (SCZ, HC; [33]).
Study protocol	Participants were recruited between 2006 and 2018 via the Dutch Bipolar Cohort (BD, HC; [4, 30]), The Outcome of Psychosis Fitness Therapy (SCZ, HC; [31]), the Spectrum (SCP, SCZ, HC; [32]), the Understanding Hallucinations (SCZ, HC; clinicaltrials.gov identifier NCT02460965), or the Simvastatin for recent onset psychosis studies (SCZ, HC; [33]).
Data collection	Participants were recruited between 2006 and 2018 via the Dutch Bipolar Cohort (BD, HC; [4, 30]), The Outcome of Psychosis Fitness Therapy (SCZ, HC; [31]), the Spectrum (SCP, SCZ, HC; [32]), the Understanding Hallucinations (SCZ, HC; clinicaltrials.gov identifier NCT02460965), or the Simvastatin for recent onset psychosis studies (SCZ, HC; [33]).
Outcomes	Connectivity strength was defined as the mean of the edge weights in the MST matrix. MST diameter, kappa, and leaf fraction were calculated to characterize global network topology (see Figure 1 and Table 2). Diameter measures the longest distance between the two most remote nodes in the network [27, 29], similar to the path length in conventional graph analysis. Kappa is a measure of diversity in nodal degree (kappa = <degree2>/<degree>). Leaf fraction quantifies the fraction of nodes in the whole network that have only one connecting edge and thereby is a measure of network integration, with higher leaf fraction indicating more integrated network topology. For regional network analyses, the degree and betweenness centrality were calculated (see Figure 1): The degree states how many edges connect to a node [27, 29]. Betweenness centrality measures how likely it is to pass a given node when connecting any two other nodes in the network. Together, these regional measures describe the nodal importance within the network.

Magnetic resonance imaging

Experimental design

Design type	resting state
Design specifications	N.A.
Behavioral performance measures	N.A.

Acquisition

Imaging type(s)	functional
Field strength	3T
Sequence & imaging parameters	3D PRESTO pulse sequence with parallel imaging (SENSE)
Area of acquisition	whole brain scan

Diffusion MRI ☐ Used ☒ Not used

Preprocessing

Preprocessing software	Images were processed using the FMRIB Software Library (FSL) version 5.0.4 [40]. For preprocessing, FEAT's default settings were used including skull stripping (BET), motion correction with MCFLIRT, spatial smoothing (5 mm kernel at full width at half maximum), and high-pass filtering (100-second cut-off).
Normalization	No global signal regression was performed as this could influence network topology analyses and group comparisons [41].
Normalization template	Time series of each voxel across the brain were extracted and averaged across 264 functional regions according to the atlas described by Power and colleagues [45].
Noise and artifact removal	A systemic motion-related bias was prevented by excluding subjects whose relative root mean square displacement

Noise and artifact removal

over all frames exceeded .2 mm or if 20 individual frames all exceeded the threshold of .25 mm [42]. ICA-AROMA was used to correct for in-scanner motion since it removes motion-related variance from the BOLD signal together with white matter and cerebral spinal fluid regressors [43, 44].

Volume censoring

A systemic motion-related bias was prevented by excluding subjects whose relative root mean square displacement over all frames exceeded .2 mm or if 20 individual frames all exceeded the threshold of .25 mm [42]. ICA-AROMA was used to correct for in-scanner motion since it removes motion-related variance from the BOLD signal together with white matter and cerebral spinal fluid regressors [43, 44].

Statistical modeling & inference

Model type and settings

Statistical analyses were performed in IBM SPSS Statistics 25. Differences in subject characteristics were tested with one-way ANOVAs and Chi-Square tests. Normality of each outcome measure per group was assessed using the Shapiro-Wilk test and Q-Q plots. The Pearson correlation between motion (relative mean displacement) and connectivity strength was calculated to assure that motion did not confound the functional connectivity measures. As main analysis, we tested for group differences in global MST measures with ANCOVAs using the complete samples with age, sex, and years of education as covariates and Tukey LSD post-hoc tests. To correct for potential type I errors, the Benjamini-Hochberg procedure was used to correct for multiple testing. As sensitivity analysis, we manually matched the groups on age since age differences might bias differences in connectivity strength and brain network topology. Exploratory post-hoc ANCOVAs using the matched groups with age, sex, and education as covariates were performed to validate global MST group differences. Additionally, Matlab R2015b (The Mathworks, Inc) was used to investigate differences in local MST topology (i.e., differences in nodal degree and betweenness centrality) between the age-matched groups using permutation tests (10,000 permutations, Monte Carlo 2-sided test, Family Wise Error adjusted). To further check for medication effects, we repeated the ANCOVAs on global MST measures and the regional permutation tests comparing BD patients with (N = 66) and without antipsychotic medication (N = 65), and BD patients with (N = 85) and without (N = 51) lithium. The significance level for all statistical tests was set at $p < 0.05$.

Effect(s) tested

Statistical analyses were performed in IBM SPSS Statistics 25. Differences in subject characteristics were tested with one-way ANOVAs and Chi-Square tests. Normality of each outcome measure per group was assessed using the Shapiro-Wilk test and Q-Q plots. The Pearson correlation between motion (relative mean displacement) and connectivity strength was calculated to assure that motion did not confound the functional connectivity measures. As main analysis, we tested for group differences in global MST measures with ANCOVAs using the complete samples with age, sex, and years of education as covariates and Tukey LSD post-hoc tests. To correct for potential type I errors, the Benjamini-Hochberg procedure was used to correct for multiple testing. As sensitivity analysis, we manually matched the groups on age since age differences might bias differences in connectivity strength and brain network topology. Exploratory post-hoc ANCOVAs using the matched groups with age, sex, and education as covariates were performed to validate global MST group differences. Additionally, Matlab R2015b (The Mathworks, Inc) was used to investigate differences in local MST topology (i.e., differences in nodal degree and betweenness centrality) between the age-matched groups using permutation tests (10,000 permutations, Monte Carlo 2-sided test, Family Wise Error adjusted). To further check for medication effects, we repeated the ANCOVAs on global MST measures and the regional permutation tests comparing BD patients with (N = 66) and without antipsychotic medication (N = 65), and BD patients with (N = 85) and without (N = 51) lithium. The significance level for all statistical tests was set at $p < 0.05$.

Specify type of analysis: ☒ Whole brain ☐ ROI-based ☐ Both

Statistic type for inference
(See Eklund et al. 2016)

Statistical analyses were performed in IBM SPSS Statistics 25. Differences in subject characteristics were tested with one-way ANOVAs and Chi-Square tests. Normality of each outcome measure per group was assessed using the Shapiro-Wilk test and Q-Q plots. The Pearson correlation between motion (relative mean displacement) and connectivity strength was calculated to assure that motion did not confound the functional connectivity measures. As main analysis, we tested for group differences in global MST measures with ANCOVAs using the complete samples with age, sex, and years of education as covariates and Tukey LSD post-hoc tests. To correct for potential type I errors, the Benjamini-Hochberg procedure was used to correct for multiple testing. As sensitivity analysis, we manually matched the groups on age since age differences might bias differences in connectivity strength and brain network topology. Exploratory post-hoc ANCOVAs using the matched groups with age, sex, and education as covariates were performed to validate global MST group differences. Additionally, Matlab R2015b (The Mathworks, Inc) was used to investigate differences in local MST topology (i.e., differences in nodal degree and betweenness centrality) between the age-matched groups using permutation tests (10,000 permutations, Monte Carlo 2-sided test, Family Wise Error adjusted). To further check for medication effects, we repeated the ANCOVAs on global MST measures and the regional permutation tests comparing BD patients with (N = 66) and without antipsychotic medication (N = 65), and BD patients with (N = 85) and without (N = 51) lithium. The significance level for all statistical tests was set at $p < 0.05$.

Correction

Statistical analyses were performed in IBM SPSS Statistics 25. Differences in subject characteristics were tested with one-way ANOVAs and Chi-Square tests. Normality of each outcome measure per group was assessed using the Shapiro-Wilk test and Q-Q plots. The Pearson correlation between motion (relative mean displacement) and connectivity strength was calculated to assure that motion did not confound the functional connectivity measures. As main analysis, we tested for group differences in global MST measures with ANCOVAs using the complete samples with age, sex, and years of education as covariates and Tukey LSD post-hoc tests. To correct for potential type I errors, the Benjamini-Hochberg procedure was used to correct for multiple testing. As sensitivity analysis, we manually matched the groups on age since age differences might bias differences in connectivity strength and brain network topology. Exploratory post-hoc ANCOVAs using the matched groups with age, sex, and education as covariates were performed to validate global MST group differences. Additionally, Matlab R2015b (The Mathworks, Inc) was used to investigate differences in local MST topology (i.e., differences in nodal degree and betweenness centrality) between the age-matched groups using permutation tests (10,000 permutations, Monte Carlo 2-sided test, Family Wise Error adjusted). To further check for medication effects, we repeated the ANCOVAs on global MST measures and the regional permutation tests

comparing BD patients with (N = 66) and without antipsychotic medication (N = 65), and BD patients with (N = 85) and without (N = 51) lithium. The significance level for all statistical tests was set at $p < 0.05$.

Models & analysis

n/a	Involved in the study
☐	☒ Functional and/or effective connectivity
☐	☒ Graph analysis
☒	☐ Multivariate modeling or predictive analysis

Functional and/or effective connectivity: wavelet coherence

Graph analysis: minimum spanning tree
